# The vaginal microbiome, papillomavirus infection, and cervical cancer: established associations in search of model systems and mechanistic answers

**DOI:** 10.1128/mbio.02677-25

**Published:** 2026-02-10

**Authors:** Gianna V. Passarelli, Sonia N. Whang, Nicole M. Gilbert, Jiafen Hu

**Affiliations:** 1 The Jake Gittlen Laboratories for Cancer Research, Pennsylvania State University, College of Medicine 12310, Hershey, Pennsylvania, USA; 2 Department of Pathology and Laboratory Medicine, Pennsylvania State University, College of Medicine 12310, Hershey, Pennsylvania, USA; 3 Penn State Cancer Institute, Pennsylvania State University, College of Medicine 12310, Hershey, Pennsylvania, USA; 4 Department of Pediatrics, Division of Infectious Diseases, Washington University in St. Louis School of Medicine 12275 , St. Louis, Missouri, USA; 5 Department of Molecular Microbiology, Washington University in St. Louis School of Medicine 12275 , St. Louis, Missouri, USA; 6 Department of Obstetrics and Gynecology, Washington University in St. Louis School of Medicine 12275 , St. Louis, Missouri, USA; 7 Center for Women’s Infectious Disease Research, Washington University in St. Louis School of Medicine 12275 , St. Louis, Missouri, USA; Albert Einstein College of Medicine, Bronx, New York, USA

**Keywords:** human papillomavirus (HPV), the mouse papillomavirus (MmuPV1), lower genital infection, vaginal microbiome, bacterial vaginosis (BV), dysbiosis, cervical pathogenesis, cervical intraepithelial neoplasia (CIN), cervical cancer, co-infection, mouse model

## Abstract

High-risk human papillomavirus (HPV) infection is the causative factor for approximately 5% of all human cancers and the leading cause of cervical cancer. High-risk HPV-associated cervical cancer still claims more than 340,000 women’s lives globally each year despite the availability of prophylactic HPV vaccines. Currently, there is no medical treatment for HPV infections and associated lesions except invasive surgical procedures. For more than a decade, numerous studies have demonstrated a correlation between certain community state types (CSTs) of the vaginal microbiome and HPV-associated infection and cancer. This review aims to provide a general overview of the most recent studies on this topic, focusing primarily on clinical data linking a *Lactobacillus*-depleted vaginal microbiome (i.e., bacterial vaginosis and CST-IV) and HPV but also describing the limited mechanistic findings in the field. Finally, a novel mouse model addressing the causative effect of the vaginal microbiome on papillomavirus-associated disease progression and cancer development is proposed.

## INTRODUCTION

## HUMAN PAPILLOMAVIRUS INFECTION AND CANCERS

Human papillomavirus (HPV) is one of the most common sexually transmitted infections worldwide, with approximately 80 million people currently infected in the United States. Almost 450 distinct HPV types have been isolated and sequenced (1). HPV is grouped into five genera (alpha, beta, gamma, mu, and nu) based on their tissue tropism. HPV, especially the high-risk types, is the causative factor for the development of cervical cancer (>99%) and a portion of other cancers, including those of the vulva (70%), vagina (70%), penis (60%), anus (>90%), oropharynx (70%), and skin (50%) (2). Cervical cancer typically develops over several years, beginning with HPV-induced dysplasia in the cervical epithelium. This dysplasia progresses to cervical intraepithelial neoplasia (CIN), a precancerous condition classified into three grades based on severity: CIN 1 (mild dysplasia), CIN 2 (moderate dysplasia), and CIN 3 (severe dysplasia or carcinoma *in situ*). CIN 1 often resolves spontaneously, especially in younger women, but persistent infection with high-risk HPV types (such as HPV 16 and 18) can lead to progression to CIN 2 and CIN 3. Without appropriate screening and treatment, CIN 3 may eventually develop into invasive cervical cancer (3). This multistep process underscores the importance of early detection and intervention through HPV vaccination and regular cervical screening. The current four HPV prophylactic vaccines have shown effective protection against up to nine HPV types (4). However, these vaccines offer no help to those who are already exposed to HPV and offer no protection against uncovered HPV types (5). Several therapeutic vaccines have shown only modest effects in the treatment of CIN 2/3 (6). No curative treatment for HPV infection and associated cervical lesions is available to date. Although it is presumed that most non-monogamous sexually active individuals will be exposed to HPV infection in their lifetime, the majority (90%) can eliminate the virus within 1–2 years post-infection (7). However, this still leaves 10% of HPV-infected individuals that experience persistent infections (8–10). The mechanisms by which intrinsic host factors or extrinsic environmental factors, including co-infections with other pathogens, collectively determine whether an infection is cleared or persists are poorly understood.

HPV is a non-enveloped, small double-stranded DNA virus with a genome of approximately 8 kb. It lacks its own replication machinery and therefore relies entirely on host cellular enzymes to complete its life cycle (11, 12). During the productive viral life cycle, two major HPV oncoproteins, E6 and E7, are involved in cell-cycle regulation and apoptosis by creating a pseudo-S phase that allows viral genome amplification in a highly differentiated cell population and play an essential role in HPV carcinogenesis (13). Other genes, including the two early genes E1 and E2, are key for viral replication and transcription. Two capsid proteins, L1 and L2, are important for viral entry, assembly, and transmission. HPV can only initiate infection by gaining access to the proliferating basal cells of the stratified epithelium through a micro-wound or micro-abrasion (11, 14). A pro-inflammatory environment can enable key mechanisms in HPV carcinogenesis: oncogene E6/E7 expression, genomic instability, viral integration, and telomerase activation (15). The intricate interplay between innate and adaptive immune responses orchestrates the clearance of HPV infection to some extent (16). However, HPV infection suppresses anti-viral activity and employs evasion tactics against immune defenses (17). Therefore, identifying pivotal immune response components and unraveling HPV’s evasive mechanisms are crucial for future HPV control efforts.

Several *in vitro* systems have been developed to study HPV infection and innate immune responses in epithelial cells, but they are inefficient in supporting the complete viral life cycle (18, 19). The field of HPV research faces two critical barriers: (i) papillomaviruses are strictly species specific, and therefore, no laboratory preclinical model is available to study HPV pathogenesis; (ii) HPV requires differentiating cells to complete its life cycle, and therefore, monolayer cells cannot be used to study the viral life cycle. A raft culture system can capture some early events, but no disease can be studied entirely *in vitro*. Additionally, it is impossible to study dysplasia and host immune responses in these epithelial *in vitro* systems (11, 20). Partially due to these challenges, the cellular and molecular mechanisms underpinning HPV biology and pathogenesis remain incompletely defined. This has become a key roadblock for the development of novel treatments for HPV and HPV-associated diseases.

## THE HUMAN VAGINAL MICROBIOME

Molecular analyses over the past decade have classified the cervicovaginal microbiome into five primary “community state types” (CSTs). Four CSTs are dominated by a single species of *Lactobacillus: L. crispatus* (CST-I), *L. gasseri* (CST-II), *L. iners* (CST-III), or *L. jensenii* (CST-V) (21). As the most abundant genus in the human vaginal microbiome, *Lactobacillus* has historically been regarded as a marker of health in the genital niche due to its ability to lower the vaginal pH and inhibit the growth of pathogenic microbes. *Lactobacillus* secretes a wide range of antimicrobial compounds, such as lactic acid, hydrogen peroxide, and bacteriocins (small proteinaceous or peptide toxins), and has immunomodulatory properties (15, 22). The exception to this is *L. iners* (CST-III), which is regarded as the least protective of the genera because it is more often present along with other vaginal bacteria that are considered non-optimal (23–25). *L. iners* (CST-III) is associated with a higher vaginal pH and regarded as less stable and more likely to transition to a polymicrobial CST (CST-IV) compared to the other *Lactobacillus* species (21, 26).

Approximately one-third (21.2 million) of women in the United States and 20%–60% of women globally (27, 28) harbor a more diverse polymicrobial vaginal community that is depleted of *Lactobacillus* with high levels of *Gardnerella*, *Fannyhessea*, *Mobiluncus*, and gram-negative anaerobes, including *Prevotella and Sneathia* (29). Over the years, different terms have been used for a polymicrobial vaginal microbiome that imply a pathogenic nature, including “non-specific vaginitis,” “dysbiosis,” “unhealthy,” and “non-optimal” (29, 30). This understanding emerged because the frequent vaginal condition called bacterial vaginosis (BV) is associated with a polymicrobial vaginal microbiome. When BV is diagnosed clinically, it is defined by having three of four Amsel criteria: thin fluid consistency, fishy odor upon potassium hydroxide treatment, elevated pH (>4.5), and the presence of exfoliated squamous epithelial cells studded with bacteria (“clue cells”) (31, 32). The gold-standard classification of BV for research studies is the Nugent score, which is based on enumeration of distinct bacterial morphotypes on Gram-stained vaginal smears (33). Recently, molecular PCR-based diagnostics that detect specific BV organisms are being developed and becoming commercially available (34–36). Even though BV is highly correlated with a polymicrobial vaginal microbiome, the presence of such a microbiome is not always associated with symptoms (37). Multiple studies have reported that healthy, asymptomatic women can harbor polymicrobial, *Lactobacillus*-deplete CSTs, including CST-IV (38). Hence, although there is a clear link between BV and CST-IV, not all instances of CST-IV can be equated with BV (21).

Irrespective of symptoms, BV or CST-IV have been associated with increased risk of adverse gynecologic outcomes and other urogenital infections, including HPV, HIV, *Chlamydia trachomatis*, *Neisseria gonorrhoeae*, urinary tract infection, endometritis, and pelvic inflammatory disease (39–46). Some instances of BV-associated infections could be due to overlapping social, behavioral, or demographic risk factors that enhance susceptibility to both (see recent review [47]). Other times, the polymicrobial vaginal microbiome could be creating an environment that is more susceptible to pathogen invasion or that otherwise enhances pathogenesis. The biochemical environment of BV/CST-IV is different from a *Lactobacillus*-dominant environment in several ways. In BV/CST-IV, the pH is increased, and there are lower levels of lactic acid. Both features are attributed to the decrease in *Lactobacillus* (15, 22). Clinically, BV is characterized by a distinctive “fishy” vaginal odor, which is caused by biogenic amines (BAs) such as putrescine, cadaverine, and trimethylamine (48). These BAs are produced by several microbes, including *Gardnerella*, *Prevotella*, *Pseudomonas*, *Enterococcus*, and *Mobiluncus* (48, 49). It has been proposed that elevated pH levels enhance the production of these BA compounds, which in turn may facilitate biofilm formation. A polymicrobial biofilm often containing *Gardnerella* and other BV-associated bacteria is visible in vaginal biopsies and on shed epithelial cells from women with BV (50, 51). This biofilm contributes to the formation of epithelial “clue cells,” a hallmark of BV diagnosis using Amsel’s criteria (52, 53). Furthermore, vaginal fluid from women with BV contains a higher number of exfoliated epithelial cells, indicating disruption of the epithelial barrier (54). Vaginal samples from women with BV display a proteomic signature of neutrophil migration and epithelial barrier disruption pathways, including elevated matrix-metalloprotease 9 (MMP9) and decreased desmoglein-1 (55). Cervicovaginal fluid, which is rich in heavily glycosylated mucins, becomes susceptible to degradation by BV-associated sialidases and proteases. These enzymes are secreted by vaginal bacteria such as *Gardnerella* and *Prevotella* but not by *Lactobacillus* species (56–59). This enzymatic activity compromises the integrity of the cervicovaginal mucus, likely contributing to the thinning of the vaginal fluid. Disruption of the mucosal barrier and mucins may result in the loss of protective properties, rendering the vaginal epithelium more vulnerable to pathogen infections, such as HPV (21), as discussed in more detail below.

## THE LINK BETWEEN THE VAGINAL MICROBIOME, HPV INFECTION, AND CERVICAL CANCER

The relationship between HPV-associated cervical cancer and vaginal microbiome has been reviewed (60–70). In general, HPV infection and disease are often linked to a more diverse and *Lactobacillus*-depleted cervicovaginal microbiome, colonized by anaerobic bacterial taxa such as species of *Gardnerella*, *Prevotella*, *Sneathia*, *Mobiluncus mulieris*, and *Megasphaera* (39, 71–78) (Table 1). As described above, these anaerobic bacterial species constitute the CST-IV vaginal microbiome and are also found in BV (29, 79). Clinical studies have examined the association between the composition of the vaginal microbiome and the incidence of HPV infection and associated cancer in different disease stages and age groups (22, 39, 73, 80–85) (Table 1). Several studies have shown that a vaginal microbiome depleted of *Lactobacillus* is associated with HPV infection, as well as HPV-associated neoplasia and cancer (Table 1). Individuals infected with HPV have displayed consistently higher abundances of specific CST-IV/BV-associated bacteria such as *Gardnerella*, *Prevotella*, *Sneathia*, and *Clostridiales* (Table 1). Conversely, the average abundance of *Lactobacillus* is significantly lower in individuals infected with HPV or with CIN, which is a precursor marker for cervical cancer (74, 86). The proportion of *Lactobacillus* was lower during both pregnancy and HPV infection when compared to the nonpregnant, HPV-negative control group (82).

**TABLE 1:** Human studies reporting an association between HPV infection and vaginal microbiome composition^*a*^

Author	Study type	Population #/country	Method/readout	Key findings	Reference
Brusselaers et al.	Systematic review and meta-analysis	101,049/multi-countries from 15 prospective studies	Gram staining; Pap smears; 16S rRNA or cpn60 gene sequencing	Vaginal dysbiosis high in HPV persistence; high-grade lesions and CIN/cancer	39
Chen et al.	Prospective study on pregnant and nonpregnant women HPV^+^/HPV^−^	135/China	16S rRNA gene fragments (V3–V4)	Increased bacterial richness and diversity and reduced *Lactobacillus* in pregnancy and HPV^+^ women. *Bifidobacterium* increases with HPV^+^, and Firmicutes increased in pregnancy but not HPV^+^	82
Dong et al.	Longitudinal study following up 0, 12, and 24 months	920/China	HR-HPV genotyping; cervical cytology; 16S rRNA gene sequencing	Women with HSIL and persistent HR-HPV such as HPV 16 infection had an abundance of *Gardnerella* and *Prevotella* and a lack of abundance in *Lactobacillus*.	81
Incognito et al.	Systematic review	2,082/from 28 articles	16S rRNA gene sequencing	Distinct levels of *L. crispat*us and *L. iners* were observed, along with variations between *Lactobacillus*-dominant and *Lactobacillus*-depleted community types	75
Kawasaki et al.	Prospective study on HPV^+^ with cervical neoplasia	248/Japan	Cervical swabs 16S rRNA gene sequencing	*Prevotella and Atopobium* were associated with SCC in younger women, while *Peptoniphilus*, *Fusobacterium*, and *Porphyromonas* were more prevalent in older women	83
Kwasniewski et al.	Prospective study on women recruited from cervical cancer screening	250/Poland	Cervical swabs 16S rRNA gene sequencing	HSIL HPV^+^ were rich in *Gardnerella vaginalis* and *L. acidophilus* in contrast to healthy women with increased *L. crispatus, L. iners*, *and Lactobacillus taiwanensis*	22
Lee et al.	Prospective study on Pap Smear samples	912/Korea	Pap smear, HPV screening, and 16S rRNA gene sequencing	Lactobacillus low in HPV^+^/CIN; Fusobacteria, including *Sneathia* spp., were a possible marker associated with HPV infection	74
Mancilla et al.	Systematic review	131,183/from 35 articles/Latinas		Out of 42 unique bacteria, *L. crispatus*, *L. iners*, *C. trachomatis*, *Prevotella* spp., Prevotella amnii, *Fusobacterium* spp*.*, and *Sneathia* spp. were enriched across multiple stages of cervical carcinogenesis in Latinas.	87
Martins et al.	Systematic review and meta-analysis	6 studies/7,119 multi -countries	HPV DNA PCR and Nugent criteria for BV	Women with BV were 2.68 times more likely to have cervical HPV infection vs those without BV	45
McKee et al.	Prospective study on HR-HPV^+^/cervical cytology samples	308/USA	Liquid cytology samples; 16S rRNA gene sequencing	HR-HPV^+^ women had higher CST -IV (e.g., *G. vaginalis*) and CST-III (e.g., *L. iners*); abnormal cervical cytology/HPV^+^ had diverse community with higher *G. vaginalis* and reduced *Lactobacillus*.	88
Mei et al.	Prospective study on women with HR-HPV persistence, clearance, and no history of any HR-HPV	100/China	HR-HPV detection; cytology; 16S rRNA gene sequencing	HR-HPV persistent and clearance groups had an increase in *Gardnerella* and lower vaginal diversity compared to the control group	89
Norenhag et al.	Systematic review and network meta-analysis	1,251/11 studies of multi-countries	HPV genotyping, 16S rRNA gene sequencing, microarray, and histology	*L. gasseri* and low *Lactobacillus* species were found among HR-HPV^+^ women and high risk of SIL/cancer. *L. iners* was associated with CIN1/2; *Prevotella, Atopobium,* and *Gardnerella* are associated with HPV persistence.	77
Peng et al.	Cross-sectional study	854/China	HPV genotyping and key microecological analysis	HPV^+^ groups showed elevated vaginal pH, hydrogen peroxide positivity, leukocyte esterase, sialidase activity, and reduced *Lactobacillus* abundance, poorer vaginal cleanliness	85
Pu et al.	Prospective study on HPV clearance in CIN patients post-LEEP	80/China	16S rRNA gene sequencing; quantitative analysis of SCFAs	*Lactobacillus* and certain anaerobes (e.g., *Prevotella bivia*) were correlated with HPV clearance post-LEEP.	84
Shi et al.	Longitudinal study-HPV clearance	85/China	Cervical swabs collected, 16S rRNA gene sequencing	HPV clearance in patients (58.9%) within 12 months; high *L. iners* negatively correlates with HPV clearance	80
Usyk et al.	Longitudinal study-two time points of cervical samples	273/Costa Rica	16S V4 rRNA gene and fungal ITS1 region sequencing	Elevation of microbial diversity and positive association of V1 *Gardnerella* with CIN2+ progression	73
Zhang et al.	Compare vaginal and oral microbiome	82/164 swabs/China	Full-length 16S rRNA gene sequencing with the PacBio platform	Oral microbial diversity exhibited an inverse pattern to that of the vaginal microbiome. Dysbiosis, including *Prevotella* and *Sneathia*, was high, but *Lactobacillus* was low in the cervical cancer group	90

^*a*^ HSIL, high‑grade squamous intraepithelial lesion; LEEP, loop electrosurgical excision procedure; SCC, squamous cell carcinoma.

A recent study demonstrated that vaginal *Lactobacilli* can provide a protective effect against pathogens that depends both on the species of the pathogen and on the *Lactobacilli* strain (91). While an abundance of *L. iners* was associated with clearance of incident high-risk HPV (HR-HPV) infections in a longitudinal study (73), many other studies reported the opposite role of *L. iners* (77). For example, a longitudinal study found that individuals with a higher abundance of *L. iners* were less likely to clear HPV infection at 12 months among patients who had non-operative treatment (80). The *L. iners-*dominated CST-III was associated with HPV persistence and progression to preinvasive and invasive lesions (92). An additional study showed that a higher presence of *L. iners* relative to *L. crispatus* was linked to a two- to threefold increased risk of HPV infection (77). Overall, a microbiota dominated by *L. crispatus* CST-I is associated with a lower risk, whereas *L. iners* is associated with a higher risk of HPV, squamous intraepithelial lesion, and cancer.

In addition to *L. iners*, the presence of high microbial diversity (CST-IV) has been associated with HPV persistence and associated dysplasia (77). Specifically, increased abundance of *Prevotella*, *Sneathia*, *Clostridiales*, and *Gardnerella vaginalis* with a reduced abundance of *Lactobacillus* was observed in HPV-infected compared to non-infected individuals (74). A similar pattern was seen in women with abnormal cervical cytology and HR-HPV infection (88). *Prevotella* spp. could be predictive of CIN2+ lesion development in cases of persistent HR-HPV infection (93). Vaginal dysbiosis was also associated with a twofold increase in risk of high-grade CIN or cancer based on data from 15 prospective studies in a systematic review and meta-analysis (39).

Other alternative bacteria were associated with HPV clearance. For example, samples clearing HPV 16 and 18 had a higher composition of *Bifidobacterium*, meanwhile samples clearing other HR-HPVs contained more *Lactobacillus*. This led investigators to assume *Bifidobacterium* may have a protective effect against HPV 16 and 18, while *Lactobacillus* has a stronger role in protection against other HR-HPVs (89). Together, these findings support the conclusion that a dominance of *L. crispatus* and several other non-*iners Lactobacilli* may contribute as a protective vaginal microbiome to prevent HPV infection, and a polymicrobial microbiome promotes infection.

### Potential mechanisms of vaginal microbiome contribution to cervical cancer

**Fig 1:**
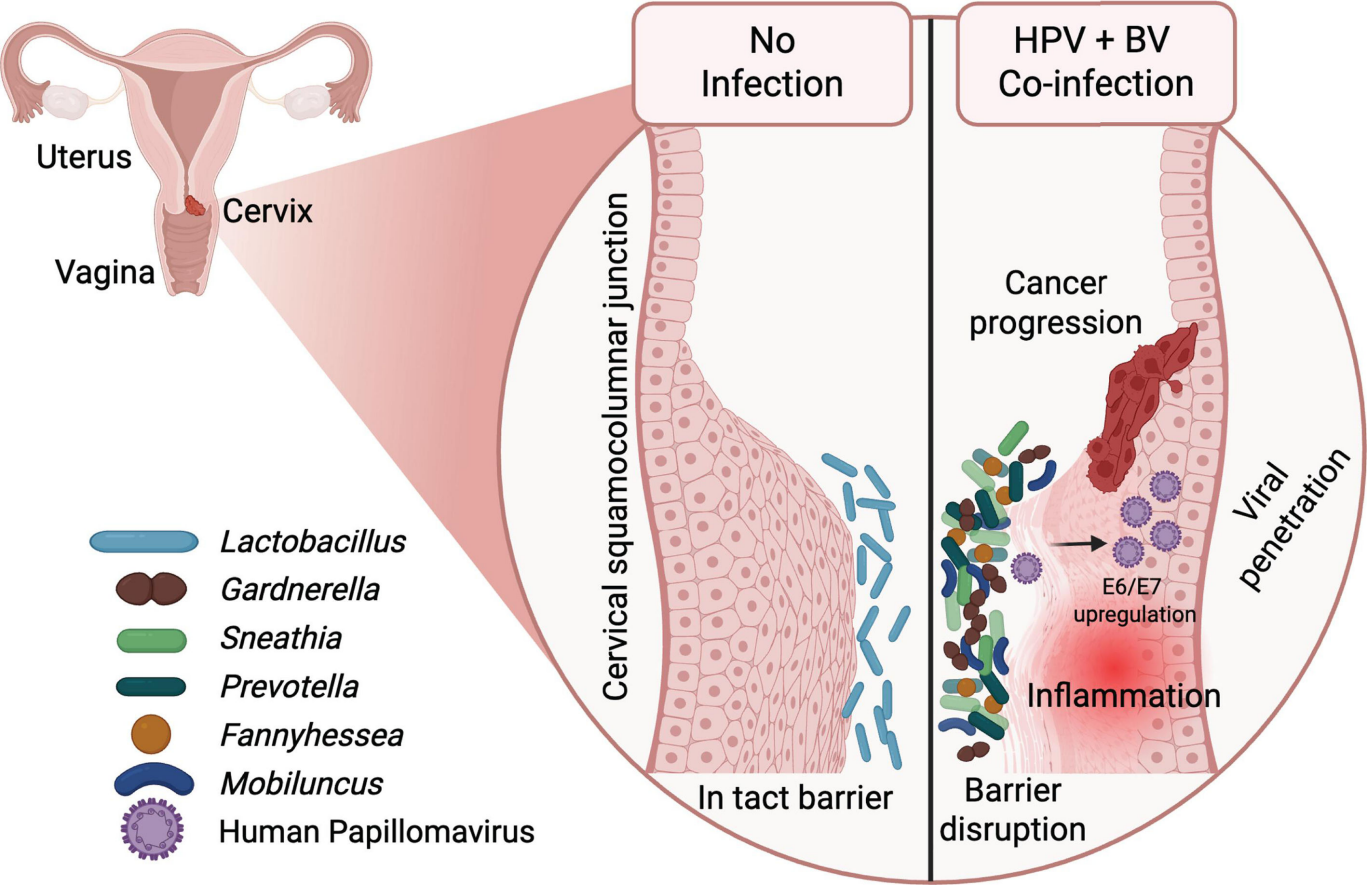
Schematic of the cervicovaginal epithelium in the context of a *Lactobacillus* dominant CST-I, CST-II, or CST-V vaginal microbiome (left) and in the context of a non-*Lactobacillus* dominant BV/CST-IV vaginal microbiome with HV infection (right). Epithelial cells and microbes are not shown at scale. Figure generated at Biorender.com.

Several mechanisms have been proposed by which a polymicrobial (CST-IV or BV) vaginal microbiome promotes HPV-associated cervical cancer (94, 95). These include mucosal disruption and immune modulation (96). HPV utilizes various mechanisms to evade immune responses to progress from infection to chronic dysplasia and cancer (97). The vaginal microbiome is also known to affect mucosal immunity in the female reproductive tract. BV-associated bacteria are reported to alter vaginal immune responses, which additionally contribute to increased susceptibility and persistence of HPV infections, key factors in cancer development (55, 77, 98). The primary targets of HR-HPV in squamous epithelia are undifferentiated keratinocytes. An *in vitro* study demonstrated that metabolites produced by *Lactobacillus* can restore E-cadherin expression and suppress MMP9 in cervical cancer cells (99). A reduction in *Lactobacillus* may lead to a pro-inflammatory environment, which can increase the expression of HPV E6 and E7 oncogenes and promote malignant cell proliferation (15). Disruption of epithelial tight junctions by pro-inflammatory bacteria can compromise the mucosal barrier, which could facilitate HPV acquisition (100) (Fig. 1). Most human studies have been retrospective, and longitudinal studies show only an association, not causation, between BV-associated bacteria and cervical cancer. The contribution of these plausible mechanisms to papillomavirus infection and tumor progression in the context of the BV/CST-IV vaginal environment remains to be defined in experimental models.

In addition to associations with the vaginal microbiome, the menstrual cycle was associated with HPV detection in reproductive-age women (101). High parity and hormonal contraceptive use have been associated with cervical cancer (102, 103). Elevated levels of endogenous estradiol have been significantly linked to persistent HR-HPV infections in young women using combined oral contraceptives (104). Cervical cancer incidence also increases with age and higher levels of follicle-stimulating hormone (105). Long-term use (>5 years) of oral contraceptives is associated with a 1.5- to 3.3-fold increased risk of cervical cancer, particularly in HPV-positive women (106, 107). Estrogen influences the amount and viscosity of vaginal secretions, the glycogen content, and the vaginal oxygen and carbon dioxide levels (108). It also significantly affects the composition of the vaginal microbiome. Low estrogen levels, as seen in prepubertal and postmenopausal women, are associated with reduced *Lactobacillus* abundance and a predominance of anaerobic bacteria (109). In contrast, the vaginal microbiome of pregnant women tends to be more stable and is typically dominated by *L. crispatus* or *L. iners* (110). Despite these studies, the interplay among sex hormones, BV-associated bacteria, and papillomavirus infection and cervical cancer remains understudied.

### HPV infection can promote a more diverse and *Lactobacillus*-depleted vaginal microbiome

Many of the clinical studies linking the vaginal microbiome to HPV are cross-sectional at a single time point and cannot pinpoint whether the microbiome is promoting HPV, or vice versa (the proverbial chicken-or-egg question). However, longitudinal studies suggest there is a two-way synergistic relationship between HPV and vaginal dysbiosis (73, 80, 81). A recent study suggested that HPV infection alters vaginal microbiome through down-regulating host mucosal innate peptides that are used by *Lactobacillus* as amino acid sources (111). These peptides are hydrolyzed, internalized, and used by vaginal *Lactobacillus* species as amino acid sources to sustain their growth and survival. Thus, the depletion of this nitrogen source by HPV could negatively impact *Lactobacillus* growth and lead to non-optimal vaginal bacteria associated with CST-IV and BV. In a longitudinal study, HPV clearance was associated with a reversal of microbial diversity and a resurgence of *Lactobacilli* (112), as well as a decrease of inflammatory cytokines (113). The potential for HPV infection to antagonize *Lactobacillus* is an intriguing potential mechanism that warrants further study.

## THE MOUSE MODEL TO STUDY PAPILLOMAVIRUS INFECTION AT THE LOWER GENITAL TRACT

HPV infects epithelial cells and requires differentiating cells to complete its life cycle (18). Therefore, *in vitro* culture systems have been very inefficient for studying HPV biology. HPV is also strictly species specific, so no preclinical model is available to study HPV infection *in vivo*. Clinical human studies can establish an association between pathogens, including viruses and bacteria, and diseases. To test the cause-and-effect relationship of these pathogens, suitable *in vitro* and *in vivo* model systems are essential. Studying human genetic and immunological determinants in populations of diverse individuals infected with a trigger pathogen has benefited from genetically modified mouse strains and novel agonists and antagonists (114). Data from mouse models provide proof of concept for developing diagnostic and therapeutic interventions to control disease. These studies, together with human studies, can also provide invaluable complementary insights into the mechanisms of immunity to infection common and specific to these two species (115).

To overcome these critical barriers, several naturally occurring animal papillomavirus models have historically played a pivotal role in understanding HPV pathogenesis (20). The mouse papillomavirus model (MmuPV1) was first reported in 2011 (116) and later demonstrated to recapitulate HPV biology at both cutaneous and mucosal tissues (117–122). Most intriguingly, MmuPV1 can infect the lower genital tract and lead to disease progression to cancer, as shown in HPV (123). We have established a non-invasive longitudinal assay to monitor viral replication using a quantitative PCR assay and disease progression using a human equivalent Pap smear cytology (123, 124). Sexual transmission was also reported (125). Like HPV, MmuPV1 infection is controlled by host immune responses (126–128). Several studies have demonstrated that long-term contraceptive use is associated with HPV persistence and cervical cancer in humans (107, 129). An early study demonstrated that estrogen plays a critical role in promoting cervical carcinogenesis in HPV-transgenic mouse models (130). We have demonstrated that administration of the hormonal contraceptive Depo-medroxyprogesterone promoted viral infection but not cancer development in the mouse PV model (131). Whether and how other contraceptives will have a similar effect or promote cancer progression in the mice remains to be determined. The MmuPV1 model is suitable for studying HPV pathogenesis and overcoming one critical roadblock to study HPV infections *in vivo*.

## THE MOUSE MODEL TO STUDY BV

The vaginal microbiome has arisen as an exciting area of study in humans. Vaginal microbiome composition has been profiled at a single time point and longitudinally in women from a wide range of ages and geographic locations, and associations with both intrinsic (age, ethnicity, menopause status, pregnancy, BMI, etc.) and extrinsic factors (sexual practices, diet, smoking, sociodemographic factors, etc.) have been described. The mouse vaginal microbiome has also been characterized and, like humans, is often dominated by a single organism. However, the dominant genera reported in conventional mice are *Staphylococcus*, *Enterococcus*, and *Rodentibacter* (132, 133). In fact, the presence of a *Lactobacillus*-dominant vaginal microbiome with an accompanying acidic vaginal fluid pH appears to be a uniquely human phenomenon that has not been observed in other mammals, even non-human primate species. In this context, the mouse vaginal microbiome mirrors a key defining feature of BV—the absence of *Lactobacillus*. Although mouse models of vaginal colonization with BV-associated bacteria are limited, the available data indicate that they can recapitulate some pathologies observed in BV in humans. Vaginal inoculation with the BV-associated bacterium *Gardnerella* strain JCP8151B reproduces several key BV phenotypes observed in women (134). Wild-type C57BL/6 mice colonized with *Gardnerella* strain JCP8151B displayed vaginal sialidase activity, sialoglycan degradation, and increased vaginal epithelial exfoliation, with the presence of clue-like cells coated with *Gardnerella* (134). Similar results of exfoliation and clue-like cells were reported by another group in the ICR outbred albino mouse strain colonized with *Gardnerella* strain KCTC5096, and *Gardnerella* strain ATCC14018 caused epithelial barrier disruption in BALB/c mice (135). BV is not associated with robust vaginal inflammation or a purulent discharge in women (136). Different patterns of inflammation have been observed in the three published *Gardnerella* colonization models. Neither *Gardnerella* JCP8151B in C57BL/6 mice nor *Gardnerella* KCTC5096 in ICR mice was associated with leukocyte recruitment into vaginal fluid or histological inflammation in vaginal tissues (134). There was a moderate increase in vaginal myeloperoxidase activity and serum IL-1β in the KCTC5096 ICR mouse model. The BALB/c mice colonized with *Gardnerella* ATCC14018 displayed substantial neutrophil recruitment that contributed to epithelial barrier disruption (55). The phenotypic differences between these mouse models could be attributed to the different mouse strains, differences in dosing or the time point analyzed (*Gardnerella* ATCC14018 was inoculated twice, the others once), or differing pathogenic potential between the *Gardnerella* strains. Phenotypic variation among *Gardnerella* isolates in expression of putative virulence factors (hemolysis, biofilm formation, and sialidase activity) has been widely reported (53, 135, 137).

The mouse model has been expanded to include co-infection with other BV bacteria or pathogens causing infections associated with BV (47, 138). The first of these incorporated the gram-negative BV-associated anaerobe *Prevotella bivia* to progress the model to a more diverse and polymicrobial BV phenotype. The phenotypes in the co-infected model demonstrated higher sialidase activity in correlation with *Gardnerella* JCP8151B than with *P. bivia-*infected mice. The presence of *Gardnerella* JCP8151B enhanced the ability of *P. bivia* to infect the uterus (57). In pregnant mice, co-infection with *Gardnerella* JCP8151B enhanced vaginal colonization and ascending uterine and placental infection by the neonatal sepsis pathogen Group B Streptococcus (139). These two studies demonstrate that *Gardnerella* can enhance the virulence of recognized reproductive tract pathogens, and mouse co-infection models are useful for investigating infections associated with BV.

## SOME LIMITATIONS OF THE MOUSE MODEL FOR TRANSLATIONAL STUDIES

While the growing body of evidence linking HPV-associated gynecological cancers to dysbiosis highlights the microbiota as a promising target for cancer prevention and therapy, notable limitations of mouse models for translational studies must be acknowledged. First, because mice do not possess a dominant *Lactobacillus*-based vaginal microbiome, the introduction of BV-associated bacteria does not accurately model the microbial transition from a *Lactobacillus* dominant to a polymicrobial state, as occurs during BV onset in women. Second, anatomical and physiological differences between human and murine reproductive tracts, such as variations in hormonal flux and the higher degree of epithelial keratinization in mice, may further limit the direct applicability of murine findings to human biology. The overall structure of the female reproductive tract differs markedly between humans and rodents. Humans have a simplex, pear-shaped uterus and a relatively long cervix (2–4 cm), whereas rodents have a bicornuate uterus with two long horns and a very short cervix. Regions where one type of epithelium replaces another (squamous metaplasia) are particularly prone to malignant transformation. Environmental factors, including viral infection, play a key role in metaplastic carcinogenesis. In humans, cervical cancers associated with HPV almost exclusively arise at the transformation zone, where metaplastic squamous epithelium replaces or overlies the original columnar epithelium of the endocervical canal. The rodent cervix, by contrast, features characteristic interdigitating mucosal folds that have no direct counterpart in humans (140). Histological evidence of endocervical glandular involvement is associated with higher rates of HPV-16 infection, multiple high-risk HPV types, and CIN2+ lesions (141). In mice, the infection is predominantly observed in the vaginal tract, which differs from the primary site of HPV-associated cervical cancer in humans. Third, although MmuPV1 and high-risk HPVs induce tumors through similar overall oncogenes, the MmuPV1 E6 and E7 proteins target distinct cellular signaling pathways (119). Notably, MmuPV1 E6 does not bind or degrade p53 via E6AP (the canonical mechanism of hrHPV E6), but instead disrupts Notch signaling through MAML1 and TGF-β signaling through SMAD2/3 (142–145). In contrast, MmuPV1 E7 binds Rb using a non-LXCXE motif (unlike the classic LXCXE motif of hrHPV E7) and uniquely targets the tyrosine phosphatase PTPN14 (146, 147). Despite these differences, MmuPV1 E6 and E7 both activate MEK/ERK signaling (148). A very recent study further showed that MmuPV1 E7 induces a gene-expression phenotype that closely resembles that of high-risk HPV infections, reinforcing the translational relevance of the MmuPV1 mouse model for studying HPV-associated pathogenesis (149). Fourth, variability in housing conditions across animal core facilities can influence the endogenous microbiome, complicating the interpretation of results across research groups. A recent study reported batch-to-batch variation in MmuPV1-infected mice even within the same facility, underscoring the microbiome’s sensitivity to environmental factors (150). Finally, it is important to consider the potential impact of epithelial injury on microbiome–virus interactions in the co-infection model. Micro-wounding is critical for HPV acquisition and infection in humans. In the mouse model, mechanical and chemical scarification (e.g., cytobrush and Conceptrol gel pretreatment) are applied to increase susceptibility in the lower genital tract. These treatments breach the mucosal barrier, trigger acute inflammation, and likely alter initial microbial translocation and immune priming, which differs markedly from natural, non-traumatic HPV acquisition in humans (151).

Despite these limitations, a co-infection mouse model remains a promising system for studying the interplay between HPV, BV, and host immune regulation during tumor progression. Several strategies can help mitigate these limitations. For instance, a comparative study using a standardized viral infection protocol across multiple animal facilities with the same mouse strain could establish a baseline for microbiome variability. This variability can be further controlled by introducing specific pathogen-free mice (152). Although dysbiosis differs between humans and mice, shared microbial metabolites may exert similar biological effects, which can be evaluated in murine models (153, 154). For both systems, the healthy cervicovaginal microbiota helps maintain cervical epithelial barrier integrity and modulates local mucosal immunity. Dysbiosis alters microbial metabolites, triggers inflammation, impairs epithelial and immune barriers, and increases susceptibility to sexually transmitted infections, including HPV persistence and disease progression (155). A recent multi-omics study showed that shifts in the vaginal microbiota are accompanied by concurrent changes in host proteomic and metabolomic profiles in mice (156). Using single-cell and spatial transcriptomics, another recent study systematically characterized morphological and gene-expression dynamics across the ovary, oviduct, uterus, cervix, and vagina throughout the mouse estrous cycle, during decidualization, and with aging (157). Our previous studies demonstrated that genital MmuPV1 infection (especially secondary infection) does not require physical scarification (124, 158); therefore, it is possible to perform MmuPV1 inoculations in a context that more closely mimics natural HPV infection in humans. Consequently, the mouse model offers valuable proof of concept and critical insights into human disease mechanisms, potentially guiding the development of novel treatments for HPV infection and associated cancers.

## THE ESTABLISHMENT OF CO-INFECTION MOUSE MODEL TO STUDY HPV-BV INTERACTION DURING TUMOR PROGRESSION

Although the correlation between microbiome and cervical cancer has been corroborated by increasing published studies (87, 90, 159), most human studies have been conducted retrospectively, and the causative relationship between BV-associated bacteria and cervical cancer could not be determined. How these BV-associated bacteria impact HPV infection and associated tumor progression is unclear. Development of a co-infection mouse model to study MmuPV1/BV interaction during disease progression in the lower genital tract would fill several significant gaps in knowledge of the impact of BV-associated bacteria on HPV. For example, (i) are BV-associated bacteria promoting papillomavirus persistence in the genital tract by altering the local immune responses, and if so, how? (ii) Which bacterial strains play a key role in enabling HPV to avoid host defenses? (iii) Which bacterial metabolites play the key role in promoting HPV-associated tumor progression? (iv) Which regulatory pathways are impacted by BV-associated bacteria in conditioning the host to tolerate HPV persistence? (v) Does HPV-associated cancer progression promote BV bacterial growth or survival to perpetuate microbial dysbiosis?

In-depth studies on the interplay between vaginal microbiome and MmuPV1-induced neoplasia in mice could lead to novel biomarkers and innovative approaches in the development of efficient therapeutic strategies for patients with cervical cancer (94). Longitudinal sampling of mice would allow monitoring various biofluids (vaginal swabs, cervicovaginal lavage/secretions, or blood) for new biomarkers and constitute important evidence usable in personalized, preventive, and predictive medicine. This is essential for improving medical care by adopting an individualized approach and reducing the economic burden of traditional healthcare in the context of 21^st^-century medicine. The establishment of mouse models for both papillomavirus infections and BV colonization lays a solid foundation for future novel co-infection models that are essential to advance our molecular understanding of the interplay between the vaginal microbiome and HPV diseases.
